# Morbidity and mortality of children aged 2–59 months admitted in the Tanzania Lake Zone’s public hospitals: a cross-sectional study

**DOI:** 10.1186/s13104-017-2818-z

**Published:** 2017-10-10

**Authors:** Kristina Lugangira, Method Kazaura, Festus Kalokola

**Affiliations:** 1 Department of Case Management, Tibu Homa Project, URC, P. O. Box 1403, Mwanza, Tanzania; 2 Department of Epidemiology/Biostatistics, Muhimbili University of Health and Allied Sciences, P. O. Box 65015, Dar es Salaam, Tanzania

**Keywords:** Case-fatality, In-patient, Morbidity, Mortality, Tanzania

## Abstract

**Background:**

There is a growing concern about child mortality especially in developing countries. The Government of Tanzania and non-governmental organizations are fighting against diseases like malaria, anaemia, diarrhoea and pneumonia that contribute extensively to child mortality. This was a hospital-based, retrospective cohort study involving 1130 under-fives (excluding neonates) being either discharged from or died in public hospitals of the Lake Zone in Tanzania. We extracted information on symptoms and signs at admission, major diagnoses and causes of death from the medical records. We applied binary logistic regression models to assess risk factors associated with in-patient under-five death.

**Results:**

The major leading morbidities include malaria (49%), anemia (37%), diarrhea (27%), pneumonia (22%) and severe acute malnutrition (21%). We found the case fatality of 74 deaths per 1000 under-five admissions. Major underlying causes of deaths were severe anaemia, severe malaria and severe pneumonia. Factors associated with in-patient death were female sex (AOR 1.7; 95% CI 1.0, 2.8) and the odds significantly decreased with increasing level of maternal education.

**Conclusions:**

Malaria remains a leading cause of admissions in hospitals among under-fives. Although the case fatality among children aged between 2 and 59 months admitted in hospitals in Lake Zone is decreasing, efforts are needed to address major causes of deaths (severe anaemia, severe malaria and severe pneumonia).

## Background

The fourth United Nation’s Millennium Development Goal advocated the reduction of under-five death by two-thirds between 1990 and 2015 [1]. There are reports of decreasing risks of child mortality globally and in some developing countries, specifically from sub-Saharan Africa including Tanzania [2–5]. Nevertheless, the gap of mortality levels among under-fives between industrialized and the less developed countries is still wide [6–8]. Major setbacks to reduce infant and child mortality include maternal and child morbidities, for example HIV and other infectious diseases like malaria and diarrhea [9–13]. In 2000, the magnitude of under-five mortality in sub-Saharan Africa was estimated at 175 per 1000 live-births [14].

In Tanzania, the under-five mortality has been decreasing over time. For example, the mortality dropped from 153.6 in 1991/92 to between 50 and 80 per 1000 live births in 2010 [15–17]. Despite the reported decreasing risks, there is variability of under-five mortality between and within regions. For example, in the Lake Zone of Tanzania, the under-five mortality was close to 170 in 1991 and decreased to about 110 per 1000 live births in 2010 [15, 16]. These risks are substantially higher than the national estimate.

Causes of under-five mortality are diverse. Literature from sub-Saharan countries indicates the major causes to be diarrhoea, malnutrition, pneumonia, malaria and anaemia; meningitis, HIV infection, gastroenteritis/dehydration, septicaemia and tuberculosis; hypoglycaemia and acute lower respiratory tract infection [18–22].

In Tanzania, like in many other developing countries, there is lack of appropriate and accurate data on specific causes of death and associated factors not only among children but also in adults [23, 24]. Also, there are some concerns regarding poor disease management, health management information and registration systems in Tanzania [25]. Correct diagnosis, proper recording and transfer of data especially on specific causes of mortality in under-fives are often not accurate leading to unreliable morbidity and mortality estimates [26].

The Lake Zone has been receiving health care support from the Ministry of Health and Social Services and its development partners. One of these partners is the USAID-supported Tibu Homa Project (THP). The ultimate goal of THP has been to improve the diagnosis and management of severe febrile illness in children under the age of 5 years in the Lake Zone of Tanzania. In this study we aimed to estimate the major causes of in-patient morbidity and case fatality among hospitalized children under the age of 5 years in the Lake Zone.

## Methods

### Study design

This was a hospital-based, retrospective cohort study. Recruitment into the study was conducted between September and November 2015.

### Study settings

The Lake Zone is situated in the North-West of Tanzania along the Lake Victoria. It comprises of six administrative regions, namely Geita, Kagera, Mara, Mwanza, Shinyanga and Simiyu. The Lake Zone has an estimated total population of about 11.8 million (26.3% of the nation’s population) of which about 11.5% are children below 5 years [16]. By the time of the study, the Zone had a total of 37 Districts (an administrative sub-division of a Region).

In Tanzania, there are public and private health care facilities. The public health care system is layered into five levels, starting with the dispensary level, the health centre, the district, regional and topped with zonal referral hospitals. These levels are differentiated by the staffing, equipment and supplies, range of services available and type and number of patients eligible for admission. Everywhere in Tanzania, including the Lake Zone hospitals, district and regional hospitals are considered referral facilities; getting patients from the lower levels (Health Centres and Dispensaries).

For the purpose of this study, the focus was on three regions (Kagera, Mara and Mwanza) that were among the regions supported by the Tibu Homa Project (THP). Therefore, the three regional hospitals were selected. Also included in the study, were three randomly selected district hospitals; one from each region. These were Ngara (Kagera Region), Tarime (Mara Region) and Magu (Mwanza Region). These health facilities are expected to have reliable services and more experienced medical staff, having advanced examination medical services (basic laboratory examinations and X-ray facilities) and improved pharmacies. Therefore, they are in better position to correctly assess causes of deaths than lower health facilities.

### Study population

The study included children aged below 5 years (excluding neonates) hospitalized during the study period. We excluded neonates because in almost all facilities there is lack of neonatal wards and these babies stayed with their mothers in female or maternity wards. We also excluded children who were referred to other facilities because it was not possible to ascertain their treatment outcomes (discharged or died). We collected data among study participants between admission until discharged or death. During the study period, information was retrieved from hospital clinical records and transcribed into study tools.

### Sample size and sampling procedure

We estimated a sample of 1155 children based on cluster sampling. Parameters used during the sample size estimation include child inpatient mortality of 60 per 1000 admissions, 95% confidence interval and a 2% margin of error. Furthermore, since the use of cluster sampling does not give equal chance to be selected, we assumed a design effect of 2. We finally adjusted the sample size taking into account non-response that was estimated to be 6%. This sample was split between six clusters. Clusters were public regional and district hospitals. One district hospital was randomly selected per region to make a total of six hospitals. Public regional and district hospitals are facilities with referral medical services. These health facilities are reliable with experienced medical staff, equipped with examination and medical services, (basic examination laboratories and X-ray facilities) and with stocked pharmacies. Based on the hierarchy, regional hospitals are superior to district hospitals. They have more specialized personnel, surgical medical care, equipment and extended capacity to admit. However, all regional and district hospitals have at least one specialist in paediatrics and they also perform laboratory services.

### Recruitment procedure

Recruitment was at death or discharge of the child when information was collected on diagnosis and cause of death. Study information was drawn from case notes after obtaining consent from biological parents or legal caregivers.

### Process

The study personnel who included, three clinicians and a nurse (staff attached to pediatric wards) were identified from the facility. These are care providers who attend children and had been previously trained by Tibu Homa Project in proper diagnosis and treatment of under-fives using national protocols and guidelines. Complete information about signs and symptoms were obtained from parents or caregivers at admission. Present and past medical histories and detailed family histories of all admitted under fives were recorded. All children were assessed for general danger signs and main symptoms according WHO guidelines. Nutritional status information was available for all children at admission. The information was recorded by measuring weight, length and mid-upper arm circumference and classified using Z-scores. Later on, this information was abridged and compiled for under-fives enrolled to the study.

Specific causes of deaths among children were obtained using patients’ clinical notes and from diagnosis made on death certificates. Most of the variables for the child were based on parent/care-givers report using face-to-face interviews. For example, age, sex, religion; relationship to the child (parents or kin caretaker), education level attained by the parent/care-giver, marital status and occupation. Education level was classified as “never in school”; “some primary” and “above primary”. Occupation was categorized as “salaried”, “unemployed”, “subsistence farming” and “self-employed/wage earners”.

Two independent death auditors reviewed patients’ files and death certificates to ascertain the cause of death using International Classification of Disease 10th Revision (ICD-10). Whenever there was no definite cause of death or when there was disagreement between death auditors, the cause of death was considered “unknown”.

### Data processing and analysis

We performed double entry of data to verify and minimize random errors so as to enhance quality of data. Then, syntax programmes and univariate analyses were performed to detect and correct inconsistencies and out-of-range values. Detected inconsistencies and out-of-range values were set to ‘missing’. Frequencies were used to get descriptive statistics. We also performed cross-tabulations to assess the associations between case fatality and pre-determined variables. These variables were included based on theory, research experience and empirical evidence. A cut-off point for a statistical significance was set at a p value of 0.05. Finally, independent factors for inpatient under-five death were determined using binary logistic regression in the multivariable model, controlling for the cluster design effect. Since under-fives were not completely randomly selected, during the analysis confidence intervals and p-values were based on robust estimation of variance by accounting for clustering of study participants within the same facility and their residence. All these procedures were performed using SPSS for Windows (Version 20).

## Results

### Description of under-fives

A total of 1130 (response rate 97.8%) study participants were included in the study; the shortfall mainly caused by low admission of under-fives in some district hospitals after the targeted study duration. Male and females were almost equally enrolled (Sex ratio, 100:100.9). Their mean age was 21.1 (SD = 14.5) months. The majority, 970 (86.5%), waited at least two days before seeking medical treatment (Table 1).

**Table 1:** Socio-demographic characteristics of children aged 2–59 months admitted in public hospitals in the Lake Zone (N = 1130)

Characteristic	Number (%)
Source of the sample (region)	
Kagera	382 (33.8)
Mara	269 (23.8)
Mwanza	479 (42.4)
Sex of the child	
Male	552 (48.8)
Female	547 (48.4)
Missing	31 (2.8)
Age (months)	
2–11	354 (31.3)
12–23	372 (32.9)
24–35	191 (16.9)
36–47	124 (11.0)
48–59	89 (7.9)
Days before seeking health care	
< 2	152 (13.5)
2–4	747 (66.1)
5 +	223 (19.7)
Missing	8 (0.7)

### Description of parents/caregivers

The majority of accompanying parent/caregiver, 975 (91.9%) were females; 1043 (95.7%) being biological parents and 954 (85.2%) having attained some primary education level and above. Those accompanying the ailing children were relatively young, mean of 28.8 (SD = 7.5) years (Table 2).

**Table 2:** Background characteristics of parents/caregivers of children aged 2–59 months admitted in public hospitals in the Lake Zone (N = 1130)

Background characteristics	Number (%)
Sex	
Male	86 (7.6)
Female	975 (86.3)
Missing	69 (6.1)
Age [mean, (SD)] years	28.8 (SD = 7.5)
Current marital status	
Never married	51 (4.6)
Married (monogamous)	827 (73.9)
Married (polygamous)	139 (12.4)
Other^a^	102 (9.1)
Education status	
Never in school	166 (14.7)
Some primary	803 (71.1)
Above primary	151 (13.3)
Missing	10 (0.9)
Employment status	
Salaried	778 (68.8)
Unemployed	213 (18.9)
Subsistence farmer	70 (6.2)
Self-employed/wage earners	58 (5.1)
Missing	11 (1.0)
Relationship to the child	
Parent	1043 (92.3)
Kin or other	47 (4.2)
Missing	40 (3.5)

^a^Widow, divorce, separate

### Morbidities among under-fives

Parents or caregivers reported on signs and symptoms of the child at admission. The major signs and symptoms that were reported included fever 995 (88.1%), vomiting 518 (45.8%), diarrhoea 397 (35.1%) and cough 384 (34.0%). The major diagnoses were malaria, 555 (49.1%), anaemia 420 (37.2%), diarrhoea 305 (27.0%), pneumonia 249 (22.0%), and malnutrition 219 (20.5%). Other reported signs and symptoms and final diagnoses are presented in Table 3. Of the malaria cases, 253 (45.6%) were also anaemic.

**Table 3:** Prevalence of morbidities among children aged 2–59 months admitted in public hospitals in the Lake Zone (N = 1130)

Symptom/diagnosis	Number (%)
Major symptoms	
Fever	995 (88.1)
Vomiting	518 (45.8)
Diarrhoea	397 (35.1)
Cough	384 (34.0)
Difficulty in breathing	189 (43.3)
Convulsions	153 (36.5)
Pallor	75 (6.6)
Body swelling	49 (4.3)
Abdominal discomfort	29 (2.6)
Other	279 (24.7)
Major diagnoses^a^	
Malaria	555 (49.1)
Anaemia	420 (37.2)
Diarrhoea	305 (27.0)
Pneumonia	249 (22.0)
Severe stunting	219 (20.5)
Moderate acute malnutrition	150 (13.7)
Urinary tract infections	113 (10.0)
Severe acute malnutrition	92 (8.4)
HIV/AIDS	32 (2.8)
Sickle cell disease	13 (1.2)
Any other	216 (19.1)

^a^One child may have multiple diagnoses. Therefore, here we present overlapping diagnoses

### Case fatality among inpatient under-fives (2–59 months)

**Fig. 1:**
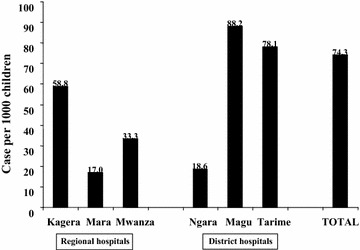
Case-fatality of children aged 2–59 months admitted in the Lake Zone’s public hospitals

During the study period, 84 deaths were recorded. This is equivalent to a case fatality of 74 (95% CI = 60.0, 91.6) per 1000 under-five admissions in the hospitals. Case fatality was generally higher among under-five inpatients admitted in district hospitals than those admitted in regional hospitals (Fig. 1). Major underlying causes of death were severe anemia, 25 (29.8%), severe malaria 24 (28.6%) and severe pneumonia 22 (26.2%). Other underlying causes of in-patient deaths are presented in Table 4.

**Table 4:** Major underlying causes of death among children aged 2–59 months admitted in public hospitals in the Lake Zone (n = 84)

Cause	Number (%)
Severe anaemia	25 (29.8)
Severe malaria	24 (28.6)
Severe pneumonia	22 (26.2)
Malnutrition	15 (17.9)
Acute waterly diarrhoea	14 (16.7)
Other	10 (11.9)

### Morbidity by survival status

Significantly more children below the age of 5 years died with major symptoms of difficulty in breathing (p = 0.007) and convulsions (p = 0.006) than among those surviving. However, there were significantly more under-fives diagnosed with anaemia (p < 0.001), pneumonia (p = 0.009) and malnutrition (p = 0.006) among those who died than those who survived. But this was a reverse for urinary tract infections; significantly more surviving under-fives were diagnosed with urinary tract infections than among those who died (p = 0.001) (Table 5).

**Table 5:** Differences in morbidities by survival status among children aged 2–59 months admitted in public hospitals in the Lake Zone (N = 1130)

Symptoms and diagnoses	Survivors (%) n = 1046	Deaths (%) n = 84	Difference (p)^a^
Major symptoms			
Fever	923 (88.2)	72 (85.7)	0.295
Vomiting	486 (46.5)	32 (38.1)	0.085
Diarrhoea	372 (35.6)	25 (29.8)	0.175
Cough	361 (34.5)	23 (27.4)	0.112
Difficulty in breathing	166 (15.9)	23 (27.4)	0.007
Convulsions	133 (12.7)	20 (23.8)	0.006
Major diagnoses			
Malaria	517 (49.4)	38 (45.2)	0.266
Anaemia	372 (35.6)	48 (57.1)	<0.001
Diarrhoea	288 (27.5)	17 (20.2)	0.091
Pneumonia	221 (22.0)	28 (33.3)	0.009
Malnutrition	214 (20.5)	28 (33.3)	0.006
Urinary tract infections	112 (10.7)	1 (1.2)	0.001
HIV	28 (2.7)	4 (4.8)	0.209

^a^p values based on Pearson’s Chi square test

### Factors associated with in-patient death

In the bivariate analysis, factors that had p-values less than 0.2 were included in the multivariable logistic regression analysis. These include sex of the child, custodian of the child, maternal education, ever hospitalized and promptness to health facility. All these variables were adjusted for location (region). In our analysis, the risk factors associated with hospital-based in-patient death were sex of the child and maternal education attainment. Females below the age of 5 years had higher odds of deaths than males (OR = 1.7; 95% CI = 1.0, 2.8). Under-fives born of mother never in school had increased odds, (OR = 14.5; 95% CI = 1.9, 111.2), as compared to those with above primary education. Similarly, children below the age of 5 years born of mothers with some primary education had increased odds of hospital-based case fatality, (OR = 11.5; 95% CI = 1.6, 84.0), as compared to those with at above primary education (Table 6).

**Table 6:** Multivariable binary logistic regression model examining factors associated with case fatality among children aged 2–59 months admitted in public hospitals in the Lake Zone (N = 1130)

Explanatory factors	Death rate^b^	OR (95% CI)^a^	
		Unadjusted	Adjusted^c^
Sex of the child			
Male	54.3	Reference	Reference
Female	91.4	1.7 (1.0, 2.8)	1.7 (1.0, 2.8)
Child’s custodian			
Both parents	69.4	Reference	Reference
One/other	105.9	1.6 (0.9, 2.8)	1.3 (0.7, 2.5)
Maternal education			
Never in school	96.4	16.0 (2.1, 122.2)	14.5 (1.9, 111.2)
Some primary	79.7	13.0 (1.8, 94.4)	11.5 (1.6, 84.0)
Above primary	6.6	Reference	Reference
Ever hospitalized			
Yes	47.8	Reference	Reference
Never	86.3	1.8 (1.1, 3.2)	1.6 (0.9, 2.9)
Reported time before seeking health care^d^			
Early (≤ 24 h)	48.1	Reference	Reference
Late (> 24 h)	92.0	2.0 (1.2, 3.3)	1.6 (0.9, 2.7)

^a^Odds Ratio (95% confidence interval)

^b^Per 1000 under-fives admissions

^c^Adjusted for location (region)

^d^Self-reporting of time interval between onset of illness and presenting to health facility

## Discussion

In this paper, we assessed morbidities, case fatality and its associated factors among children under the age of 5 years admitted in public hospitals in the Lake Zone in Tanzania. The five leading major morbidities include malaria (49%), anaemia (37%), diarrhoea (27%) pneumonia (22%) and severe acute malnutrition (21%). Many under-five in-patients present with fever, vomiting, diarrhoea and coughing. Previous reports suggest the same leading morbidities among under-fives in developing countries including Tanzania [12, 27–29]. Furthermore, the leading causes of hospital admissions among under-fives and adults have been fever, vomiting, diarrhoea and coughing [3, 12, 30, 31].

The prevalence of malaria in this study is extremely high probably due to a high malaria infection during the rainy season (the time we conducted this survey). Also, the high prevalence maybe explained by hospital data that are more likely to over-estimate the prevalence.

Despite the decline in mortality, major underlying causes of in-patient death risks in this study were severe anaemia, severe malaria and severe pneumonia. Data from sub-Saharan and Asian countries indicate malaria, diarrhoea, pneumonia, anaemia and malnutrition as some of the major causes of deaths among children below the age of 5 years [3, 18, 32, 33]. However, severe anaemia as a cause of death from this study may be linked with severe malaria.

Our results show females having increased odds of in-patient case fatality than their male counterparts. The differences in mortality by sex between developed and developing countries has not been studied and the link between mortality and sex has not been fully established anywhere. But in Uganda, the Demographic and Health Survey reported females under the age of 5 years as having an advantage of survival over males [34]. If the reported reason of culture in viewing a girl as a source of wealth, this could be the opposite from the study area that considers inheritance of family wealth by males and *patrilineage* to perpetuate the family’s name.

## Limitations

This study has several potential limitations. First, on the one hand this was a hospital-based study with a high possibility of selectivity bias because under-fives presenting to hospitals is dependent on health seeking behavior of the parent/caregiver. In turn, this is likely to over-state the mortality level in the study area. On the other hand, exclusion of neonates in the in-patient children aged below 5 years may have under-stated the level of mortality. Nevertheless, one among other advantages of the study is to confirm the diagnoses that may be relevant for control of diseases. Second, face-to-face interviews were used rather than a self-administered tool. In using this technique, it is possible to introduce information bias because respondents might have been offering socially desirable answers. Third, multiple diagnoses of morbidities that were likely to happen in our study, may not precisely show the distribution of morbidities among study participants. Fourth, although the study tried to examine independent factors associated with case fatality, the selected factors were not exhaustive and missed several social factors such as socio-economic and cultural characteristics that may be related to mortality. Also, asking about past events relies on respondent’s memory which may be flawed. Therefore, recall bias cannot be ruled out.

## Conclusions

The current study offers quality data on hospital diagnoses and deaths based on improved diagnoses and treatment of under-fives in the Lake Zone. While malaria remains a leading cause of admissions in hospitals among study participants in this study, the majority have other morbidities that include anemia, pneumonia, diarrhea diseases and malnutrition. In this study, the facility-based in-patient case-fatality is 74 per 1000 under-five admissions. The major underlying causes of deaths are severe anemia, severe malaria, severe pneumonia and malnutrition. Factors associated with mortality are maternal education and females under the age of 5 years have increased odds of case fatality than their male counterparts.

Results from this study suggest that there is need to revised interventions to address causes of deaths in children aged below 5 years in the Zone. Also, more data are required to further assess the variations in causes of under-five deaths in the Lake Zone.

## Abbreviations

AOR: Adjusted Odds Ratio
CI: confidence interval
HIV: Human Immunodeficiency Virus
OR: Odds ratio
SD: standard deviation
THP: Tibu Homa Project
USAID: United States Agency for International Development
WHO: World Health Organization
